# Optimizing midlife metabolic syndrome thresholds for dementia: a prospective study of two UK population-based cohorts

**DOI:** 10.1186/s13195-025-01732-8

**Published:** 2025-04-23

**Authors:** Sam Vidil, Archana Singh-Manoux, Benjamin Landré, Aurore Fayosse, Séverine Sabia, Marcos D. Machado-Fragua

**Affiliations:** 1 Epidemiology of Ageing and Neurodegenerative diseases (EpiAgeing), Université Paris Cité, Inserm U1153, Paris, France; 2 Faculty of Brain Sciences, University College London, Alexandra House, 17 Queen Square, London, WC1N3AR UK

**Keywords:** Cardiometabolic risk factors, Metabolic syndrome, Optimal thresholds, Dementia, Midlife

## Abstract

**Background:**

The concept of metabolic syndrome (MetS) was developed to identify individuals at higher risk of type 2 diabetes and cardiovascular disease, but its relevance for dementia remains unclear. We examined MetS in midlife for association with late-onset dementia, focusing on the thresholds of MetS components that carry risk for dementia.

**Methods:**

MetS components (waist circumference, blood pressure, triglycerides, HDL-C, and fasting glucose) were measured on 6,137 white participants < 60 years from the Whitehall II (WII) cohort study. A changepoint method in time-to-event analyses was used to identify optimal thresholds, and those exhibiting better performance for dementia were retained to develop a revised MetS definition. Results were validated on 171,886 participants in the UK Biobank (UKB) study.

**Results:**

Over a median follow-up of 22.6 years in WII and 13.8 years in UKB, 522 and 418 late-onset dementia cases were recorded, respectively. Optimized thresholds for triglycerides and fasting glucose performed better than original MetS thresholds in WII, and were used to develop a revised MetS definition. The MetS scale had a linear association with dementia, and 1-component increment (range 0 to 5) was associated with higher dementia risk using the revised MetS definition (HR, 95% CI: 1.11, 1.03–1.19) but not the original MetS definition (HR, 95% CI: 1.06, 0.98–1.14) in WII. In UKB, the revised MetS definition exhibited better performance for dementia risk than the original definition (p for HR comparison < 0.01).

**Conclusions:**

MetS in midlife is potentially an important target for dementia prevention. However, the thresholds for triglycerides and glucose that carry risk need to be tailored specifically for dementia.

**Supplementary Information:**

The online version contains supplementary material available at 10.1186/s13195-025-01732-8.

## Background

The ageing of populations worldwide is projected to lead to a rapid increase in people living with dementia [1]. There is considerable research that aims to identify modifiable risk factors for dementia [1] for the development of prevention and intervention strategies. Cardiometabolic risk factors have attracted attention due to their potential role in dementia pathogenesis and their modifiability through targeted interventions [2–4]. 

Much of the research on cardiometabolic risk factors and dementia considers them one at a time, besides exceptions such as the metabolic syndrome (MetS) [5, 6] that includes a constellation of interrelated cardiometabolic abnormalities (central obesity, elevated blood pressure, dyslipidaemia, and elevated fasting glucose). MetS was developed primarily for outcomes such as type 2 diabetes and cardiovascular disease (CVD) [5, 7] but in recent research it has been used to examine associations with dementia because several of its individual components have been consistently associated with a higher risk of dementia [1, 8], particularly when measured in midlife [1, 9]. 

Several studies [10–13], including a meta-analysis in 2019 of longitudinal studies [14], did not find an association between MetS and dementia. One possible explanation of these findings is that many studies were not based on participants who were middle-aged at assessment of MetS, and the follow-up was < 10 years [10–13]. Another possibility is that the thresholds used to define high risk for each component of MetS may not be optimal for dementia. Previous research suggests alternative thresholds for midlife cardiometabolic risk factors in relation to cognitive outcomes [15, 16]. Tailoring thresholds of each MetS component specifically for dementia may provide more accurate and meaningful insights into the association between MetS and dementia.

The aim of our study was to first identify the optimal threshold for each component of MetS measured in midlife in relation to dementia at older ages, and then examine their individual and combined (as in MetS) associations with incidence of dementia over a 31-year follow-up. Optimal MetS thresholds were identified and evaluated in the Whitehall II (WII) study and then validated in the UK Biobank (UKB) study.

## Methods

### Study population

Primary analyses were based on WII data, an ongoing cohort study established in 1985–1988 on 10,308 (6,895 men and 3,413 women) London-based government department employees, aged 35–55 years [17]. Thereafter, seven follow-up clinical examinations have taken place approximately every 4–5 years. Linkage to electronic health records of the UK National Health Service (NHS) was used to obtain data over the follow-up. Written informed consent from participants and research ethics approvals were renewed at each wave; the latest approval was from the University College London Hospital Committee on the Ethics of Human Research, reference number 85/0938.

As a validation cohort, data were drawn from the UK Biobank study, a population-based cohort study on over half a million participants aged 40–69 years, recruited between 2006 and 2010 [18]. At baseline, participants completed a computer-based questionnaire and underwent a clinical examination [18]. All participants gave written consent for participation, and the National Information Governance Board for Health and Social Care and the National Health Service North West Centre for Research Ethics Committee approved the study (reference number 11/NW/0382).

### MetS components

In WII, MetS components measured at the 1991–1993, 1997–1999, 2002–2004, and 2007–2009 waves were used to extract measures in midlife for each participant, defined here as < 60 years (range 40 to 59.9 years). When a participant had several measurements before age 60, the measurement closest to 55 years was chosen. MetS components were waist circumference, systolic and diastolic blood pressure, triglycerides, high-density lipoprotein cholesterol (HDL-C), and fasting glucose [6]. The smallest circumference at or below the costal margin was taken to measure waist circumference. Systolic and diastolic blood pressure were the mean of two measurements using a Hawksley random zero sphygmomanometer (1991–1993 and 1997–1999) and OMRON HEM 907 (2002–2004 and 2007–2009) with the participant in a sitting position after five minutes of rest. Venous blood samples were taken after at least five hours of fasting, and serum obtained after centrifugation was refrigerated at 4 °C and assayed within 72 h of the blood draw. Fasting glucose was measured using the glucose-oxidase method. Use of medication was reported by participants, coded using the British National Formulary. It included lipid-modifying drugs, antihypertensive drugs, and glucose-lowering drugs.

In UKB, MetS components were measured at baseline (2006–2010) in participants < 60 years (range 38 to 59 years). All MetS components were the same as in WII except fasting glucose. In UKB, circulating glucose levels were obtained from non-fasting blood samples and are likely to be affected by recent food intake. Therefore, glycated haemoglobin (HbA1c) was used for assessing glucose levels. Thresholds for glycated haemoglobin were calculated to be equivalent to fasting glucose values, following American Diabetes Association recommendations and using standardized formulas [19, 20]. 

MetS classification was based on the presence of three or more components, with the original MetS definition being: elevated waist circumference (≥ 102 cm in men and ≥ 88 cm in women); blood pressure (systolic ≥ 130 mmHg and/or diastolic blood pressure ≥ 85 mmHg, or use of antihypertensive drugs); elevated triglycerides (≥ 150 mg/dL (1·7 mmol/L), or use of lipid-modifying drugs); low HDL-C (< 40 mg/dL (1·0 mmol/L) in men and < 50 mg/dL (1·3 mmol/L) in women, or use of lipid-modifying drugs); fasting glucose (≥ 100 mg/dL (5·6 mmol/L) or use of glucose-lowering drugs in WII; HbA1c (≥ 32.6 mmol/mol) or use of glucose-lowering drugs in UKB) [6]. 

### Dementia

Dementia cases were identified in both cohorts from the National Hospital Episode Statistics (HES) dataset using the unique NHS identification number and ICD-10 codes F00-F03, F05·1, G30, and G31. Ascertainment of all-cause dementia using the HES data has a sensitivity and specificity of 78·0% and 92·0%, respectively [21]. The sensitivity in our study is likely to be higher as we also used the National Statistics Mortality Register (both cohorts), and the Mental Health Services Data Set (only in WII) to improve dementia case identification. The date of dementia onset was set as the first record of dementia diagnosis using all three data sets. Data on dementia ascertainment were available until 01/03/2023 in WII, and in UKB until 31/10/2022, 31/08/2022, and 31/05/2022 in England, Scotland, and Wales, respectively.

### Covariates

Sociodemographic covariates included age, sex, education (university or higher degree, secondary school, and lower secondary school or less), and marital status (married or cohabiting, single, divorced or separated, and widowed; living alone in UKB). Health-related behaviors included smoking status (never-smoker, ex-smoker, and current smoker), alcohol consumption (no consumption, 1–14 units/week, and > 14 units/week), fruit and vegetable consumption (less than daily, once a day, and twice or more a day), and time spent in moderate and vigorous physical activity in hours per week in WII (metabolic equivalent of task (METs) in minutes per week in UKB). For every participant, covariates were extracted from the same wave as data on MetS components.

### Statistical analyses

Participants who did not consent to linkage to electronic health records, had incomplete data on MetS components or covariates, or prevalent dementia at the start of follow-up were excluded from the analyses. As our focus was on late-onset dementia, onset of dementia at < 65 years were also excluded. In addition, as interactions between diastolic blood pressure and waist circumference with ethnicity were found in WII, and given that thresholds for cardiometabolic components might be race-specific, non-white participants were excluded from all the analyses as this group was not large enough to pursue stratified analyses.

Participants were followed from the measure of MetS to the date of record of dementia, death or end of follow-up (01/03/2023 in WII, and 31/10/2022, 31/08/2022, and 31/05/2022 in England, Scotland, and Wales, respectively, in UKB), whichever came first. The analyses consisted of three steps. First, we used the changepoint method proposed by Contal and O’Quigley [22] to identify optimal thresholds in the association with dementia for each MetS component in WII. This involved maximizing the log-rank statistic along the continuous measure of each MetS component to identify the value (optimal threshold) where the difference between dementia cases and non-cases was the greatest. The Q statistic, based on a distribution on the supremum of the absolute value of a Brownian bridge, corresponding to a Kolmogorov-Smirnov distribution, was used to test the null hypothesis (the chosen optimal threshold is not associated with incident dementia). A p-value < 0·05, corresponding to a Q Statistic > 1.358 (95th percentile of a Kolmogorov-Smirnov distribution), was used to determine whether the threshold identified was relevant for incident dementia. The optimal thresholds were calculated using the *survMisc* package in R version 4.0.3 (R Core Team).

In the second step, participants were classified as having low or high risk based on the optimal thresholds identified in the previous step and also using the original MetS thresholds [6] for all five components in WII. To be consistent with the definition of MetS, participants using medication for a component were classified as high-risk individuals [6]. Then cause-specific Cox regression, with age as the timescale and analyses adjusted for sex, education, marital status, and birth cohort in 5-year groups (model 1) was used to examine the association between each MetS component (separate models) and the incidence of dementia, with the reference being participants at low risk. Subsequent analyses were adjusted for health-behaviors (smoking, alcohol consumption, fruit and vegetable consumption, and physical activity; model 2). The hazard ratio (*survcomp* package in R) and the C-statistic estimates from the original and optimized thresholds, with incident dementia as the outcome, were then formally compared. We retained for the next analyses the identified optimal thresholds that exhibited better performance (p-value for HR comparison < 0.10, and HR from optimized threshold > HR from original threshold) for incident dementia compared with original thresholds for MetS components.

In the third step, a revised definition of MetS was built by substituting the retained optimal thresholds for the original thresholds. Then, the association between MetS using the original definition (≥ 3 cardiometabolic components using original thresholds) was compared with the revised MetS definition for the risk of incident dementia using Cox regression. We also modelled MetS (original and revised definition) as a continuous variable (range 0 to 5) by summing all components and estimated the risk of dementia for a 1-point increment.

### Additional analyses

To examine the robustness of our results several additional analyses were undertaken. *One*, comparisons between the original and the revised definition of MetS were repeated in UKB for validation of our results.

*Two*, all MetS components are dichotomized in the original method but to examine if this is the case for dementia we tested non-linearity in associations by comparing a linear model (continuous exposure) with a restricted cubic spline model using the Akaike Information Criterion (AIC), Bayesian Information Criterion (BIC), and the loglikelihood ratio test in WII.

*Three*, association of MetS definition (original vs. revised definition) with incident dementia was repeated using inverse probability weighting to account for missing data in both cohorts. This involved calculating the probability of being included in the analytical sample using logistic regression that included demographic, socioeconomic, behavioural factors, as well as cardiometabolic risk factors, morbidities including dementia and mortality over follow-up, and stepwise-selected interactions between covariates. The inverse of these probabilities was used as weights in Cox regression.

*Four*, as cardiovascular disease (CVD, defined as stroke (ICD-10 codes I60–64), coronary heart disease (ICD-10 codes I20–25), and/or heart failure (ICD10 code I50)) may affect the association between cardiometabolic risk factors and the incidence of dementia, we repeated analyses comparing both definitions of MetS by excluding prevalent cases of CVD at the baseline of our analyses in both cohorts.

*Five*, the large sample size in UKB allowed us to undertake additional sex-stratified analyses to examine whether the association between the original and the revised MetS definition with the risk of dementia was similar in men and women.

All analyses besides calculation of thresholds and comparison of hazard ratios were undertaken using Stata version 16.1 (StataCorp). A two-sided p value < 0·05 was considered statistically significant.

## Results

Of the 10,308 participants recruited to the Whitehall II study in 1985–1988, 10 did not consent to linkage to electronic-health records, 159 (1.5%) died before age 60, 1,698 (16.5%) did not have data before age 60, 12 (0.1%) had dementia onset < 65 years, and 634 (6.2%) were of non-white ethnicity. In addition, 1,636 (15.9%) and 22 (0.2%) participants with missing data on MetS components and covariates respectively were also excluded, leading to a sample of 6,137 participants (mean age = 55.1 (standard deviation = 2.9) years) with data on MetS components and free of dementia at the MetS measure (Additional File 1: Figure S1). Corresponding figures in UKB (validation cohort) are also provided in Additional File 1: Figure S1. The median (IQR) of follow-up was 22.6 (19.1, 27.7) years in WII and 13.8 (13.0, 14.4) in UKB, and 522 and 418 incident dementia cases were recorded over the follow-up, respectively. Comparison of the baseline characteristics of participants excluded from the analyses with those in the analyses are shown in Additional File 1: Table S1. The characteristics common to both studies were that excluded participants were more likely to be women, and have a low education level.

Table 1 shows participants’ characteristics according to dementia status at the end of follow-up in WII and UKB. Compared to participants who did not develop dementia over the follow-up, participants with incident dementia at the end of the follow-up in both cohorts were older, had a lower educational level, and were more likely to be single or living alone. In addition, participants with dementia in both cohorts had a higher mean diastolic blood pressure at the start of the follow-up.

**Table 1:** Characteristics of participants at baseline, overall and according to dementia status at the end of follow-up in the Whitehall II and UK biobank cohort studies

	WHITEHALL II					UK BIOBANK			
	**Total** **Population**	**Dementia**				**Total** **Population**	**Dementia**		
		**No**	**Yes**	**p**			**No**	**Yes**	**p**
N	6,137	5,615	522			171,886	171,468	418	
Age, M(SD)	55.1 (2.9)	55.0 (2.9)	56.2 (2.5)	< 0.001		50.7 (5.6)	50.7 (5.6)	57.1 (1.9)	< 0.001
Sex, women	1,756 (28.6)	1,575 (28.1)	181 (34.7)	0.001		92,444 (53.8)	92,266 (53.8)	178 (42.6)	< 0.001
Education, low	2,690 (43.8)	2,397 (42.7)	293 (56.1)	< 0.001		12,978 (7.6)	12,889 (7.5)	89 (21.3)	< 0.001
Marital status, single^a^	870 (14.2)	797 (14.0)	83 (15.9)	0.24		27,541 (16.0)	27,450 (16.0)	91 (21.8)	0.001
Smoking, current smokers	710 (11.6)	645 (11.5)	65 (12.5)	0.76		19,995 (11.6)	19,933 (11.6)	62 (14.8)	0.01
Alcohol, moderate drinkers	3,346 (54.5)	3,075 (54.8)	271 (51.9)	< 0.001		62,633 (36.4)	62,510 (36.5)	123 (29.4)	< 0.001
Poor diet^b^	1,884 (30.7)	1,706 (30.4)	178 (34.1)	0.003		7,393 (4.3)	7,380 (4.3)	13 (3.1)	0.49
PA, h/week, M(SD)^c^	3.45 (3.58)	3.46 (3.55)	3.29 (3.87)	0.02		2,591 (2,717)	2,591 (2,716)	2,626 (3,184)	0.80
Prevalence of CVD	287 (4.7)	266 (4.7)	21 (4.0)	0.46		4,120 (2.4)	4,079 (2.4)	41 (9.8)	< 0.001
Cardiometabolic components									
Waist Circumference, M(SD)									
Men	91.6 (10.3)	91.7 (10.3)	90.0 (10.1)	0.003		96.0 (11.3)	96.0 (11.3)	98.7 (13.3)	< 0.001
Women	79.4 (12.3)	79.4 (12.4)	79.3 (11.7)	0.95		83.1 (12.4)	83.1 (12.4)	85.4 (12.1)	0.02
Systolic blood pressure, M(SD)	123.0 (14.9)	123.0 (14.9)	123.4 (14.7)	0.55		133.3 (17.1)	133.3 (17.1)	140.7 (20.4)	< 0.001
Diastolic blood pressure, M(SD)	77.6 (10.5)	77.5 (10.5)	79.6 (9.7)	< 0.001		82.1 (10.2)	82.1 (10.2)	83.6 (11.1)	0.002
Triglycerides, M(SD)	1.45 (0.97)	1.45 (0.98)	1.46 (0.88)	0.84		1.70 (1.06)	1.70 (1.06)	1.91 (1.18)	< 0.001
HDL-C, M(SD)									
Men	1.39 (0.36)	1.39 (0.36)	1.38 (0.37)	0.78		1.28 (0.30)	1.28 (0.30)	1.28 (0.34)	0.97
Women	1.76 (0.45)	1.77 (0.45)	1.68 (0.46)	0.01		1.60 (0.37)	1.60 (0.37)	1.60 (0.43)	0.92
Fasting glucose, M(SD)^d^	5.30 (0.95)	5.29 (0.95)	5.33 (1.01)	0.48		34.9 (6.0)	34.9 (6.0)	38.4 (10.8)	< 0.001

M: mean; SD: standard deviation; PA: moderate-vigorous physical activity; CVD: cardiovascular disease (stroke, coronary heart disease, and heart failure); HDL-C: high density lipoprotein-cholesterol. Data are n (%), unless otherwise specified

^a^Living alone in UK Biobank

^b^Poor diet defined by consumption of fruit and vegetables less than daily

^c^Metabolic equivalent of task (MET; minutes per week) in UK Biobank, M(SD)

^d^Glycated haemoglobin in mmol/mol in UK Biobank, M(SD)

Table 2 shows the original and optimal (with dementia as the outcome, using Contal and O’Quigley’s method [22]) thresholds for cardiometabolic components of MetS in WII. No statistically significant optimal threshold, indicating that such a threshold does not exist, was found for any MetS component. Although not statistically significant, all components except triglycerides had thresholds identified by this method that were more conservative than the original thresholds for MetS.

**Table 2:** Optimal thresholds of cardiometabolic risk factors for dementia risk as compared to original thresholds in the Whitehall II cohort study^a^

			Contal & O’Quigley’s method		
	Original thresholds		Optimal thresholds^b^	Q statistic^c^	*p*-value^c^
Waist circumference					
Men	≥ 102 cm		≥ 96 cm	0.97	0.31
Women	≥ 88 cm		≥ 79 cm	1.20	0.11
Systolic blood pressure	≥ 130 mmHg		≥ 129 mmHg	1.04	0.23
Diastolic blood pressure	≥ 85 mmHg		≥ 78 mmHg	0.37	0.99
Triglycerides	≥ 1.70 mmol/L		≥ 2.13 mmol/L	0.72	0.67
HDL-C					
Men	< 1.0 mmol/L		< 1.46 mmol/L	0.65	0.79
Women	< 1.30 mmol/L		< 1.41 mmol/L	1.03	0.24
Fasting glucose	≥ 5.60 mmol/L		≥ 5.20 mmol/L	0.87	0.44

HDL-C: high-density lipoprotein cholesterol

^a^N dementia cases/Total: All participants: 522/6137; Men: 341/4381; Women: 181/1756

^b^Optimal thresholds estimated with Contal & O’Quigley’s method using a cause-specific Cox regression hazard model adjusted for age, sex, education, marital status, and birth cohort (5-year groups)

^c^p-value for difference in dementia incidence between the low- and high-risk groups, defined using Contal & O’Quigley’s method or optimal threshold; a significant result corresponds to a Q statistic > 1.358 (95th percentile of a Kolmogorov-Smirnov distribution)

The associations of cardiometabolic components, using the original and suggested optimal (Contal and O’Quigley’s method [22]) thresholds, with incident dementia in WII are shown in Table 3. In the fully adjusted model (Model 2) only waist circumference (HR 1.29; 95% CI 1.01, 1.64) and HDL-C (HR 1.30; 95% CI 1.03, 1.64) were associated with higher risk of incident dementia using the original thresholds. Using the optimal thresholds, waist circumference (HR 1.28; 95% CI 1.06, 1.54) and fasting glucose (HR 1.20; 95% CI 1.00, 1.43) were associated with higher risk of dementia. Formal comparison of the hazard ratios for the original and optimal thresholds showed triglycerides and HDL-C to be significantly different (*p* < 0.05), with optimized thresholds for triglycerides exhibiting better performance for dementia and those for HDL-C showing worse performance. Optimized thresholds for fasting glucose also tended to have better performance for dementia than original thresholds (*p* = 0.08). Therefore, optimized thresholds for triglycerides and fasting glucose were retained to develop a revised MetS definition. In addition, model fit statistics suggested better fit for the linear model in associations of all MetS components with dementia (Additional File 1: Table S2 and Figure S2), except for waist circumference in men, which showed better fit for the cubic spline model.

**Table 3:** Comparison of original and optimal threshold of MetS components in the association with incident dementia in the Whitehall II cohort study^a^

		*N* dementia cases/Total			HR (95% CI)		*p*-value for HR comparison^d^	C-statistic (95% CI)	*p*-value for C-statistic comparison^e^
		Reference group	High-risk group		Model 1^b^	Model 2^c^			
**Median follow-up 22**.**6 (IQR 19.1**,** 27.7) years**									
Elevated waist circumference									
	Original threshold: ≥102 (≥ 88) cm (women)	440/5,068	82/1,069		**1.32 (1.04**,** 1.68)**	**1.29 (1.01**,** 1.64)**	0.45	0.571 (0.543, 0.599)	0.46
	Optimal threshold (Contal & O’Quigley): ≥96 (≥ 79) cm (women)	347/4,006	175/2,131		**1.32 (1.09**,** 1.59)**	**1.28 (1.06**,** 1.54)**		0.575 (0.547, 0.603)	
Elevated blood pressure^f^									
	Original threshold: ≥130 (≥ 85) mmHg systolic (diastolic)	284/3,437	238/2,700		1.05 (0.88, 1.25)	1.04 (0.87, 1.24)	0.29	0.574 (0.546, 0.602)	0.97
	Optimal threshold (Contal & O’Quigley): ≥129 (≥ 78) mmHg systolic (diastolic)	182/2,517	340/3,620		1.09 (0.91, 1.31)	1.08 (0.90, 1.29)		0.574 (0.546, 0.602)	
Elevated triglycerides^g^									
	Original threshold: ≥1.70 mmol/L	369/4,270	153/1,867		1.06 (0.88, 1.28)	1.02 (0.84, 1.24)	**0.01**	0.573 (0.545, 0.601)	0.95
	Optimal threshold (Contal & O’Quigley): ≥2.13 mmol/L	470/5,555	52/582		**1.27 (1.02**,** 1.58)**	1.23 (0.98, 1.53)		0.573 (0.546, 0.601)	
Low HDL-C^g^									
	Original threshold: <1.0 (< 1.30) mmol/L (women)	443/5,256	89/881		**1.31 (1.04**,** 1.65)**	**1.30 (1.03**,** 1.64)**	**0.04**	0.575 (0.547, 0.603)	0.43
	Optimal threshold (Contal & O’Quigley): <1.46 (< 1.41) mmol/L (women)	252/2,909	270/3,228		1.07 (0.89, 1.29)	1.05 (0.87, 1.27)		0.571 (0.543, 0.599)	
Elevated fasting glucose^h^									
	Original threshold: ≥5.60 mmol/L	406/4,726	116/1,411		1.05 (0.86, 1.30)	1.05 (0.85, 1.29)	0.08	0.574 (0.546, 0.602)	0.78
	Optimal threshold (Contal & O’Quigley): ≥5.20 mmol/L	233/2,963	289/3,174		**1.20 (1.00**,** 1.43)**	**1.20 (1.00**,** 1.43)**		0.575 (0.546, 0.604)	

MetS: metabolic syndrome; HR: hazard ratio; CI: confidence interval; IQR: interquartile range; HDL-C: high density lipoprotein-cholesterol;

^a^ The reference group for each component was composed of participants classified as being at low risk on that component

^b^ Model 1: analysis adjusted for age (as time-scale), sex, education, marital status, and birth cohort (5-year groups)

^c^ Model 2: model 1 and adjustment for health-related behaviors (smoking, alcohol consumption, consumption of fruits and vegetables, and physical activity)

^d^ Using original threshold as the reference

^e^ Using original threshold as the reference

^f^ Defined with the corresponding threshold for systolic or diastolic blood pressure or use of antihypertensive drugs

^g^ Defined with the corresponding threshold or use of lipid-modifying drugs

^h^ Defined with the corresponding threshold or use of glucose-lowering drugs

Table 4 shows MetS (≥ 3 out of 5 cardiometabolic components) not to be associated with higher risk of dementia using the original (HR 1.15; 95% CI 0.91, 1.44; model 2) or the revised definition (including optimized thresholds for triglycerides and fasting glucose; HR 1.22; 95% CI 0.98, 1.52 for model 2) in WII, with the difference in HRs not being statistically significant (p-value = 0.19). When MetS scale (0 to 5 components) was used as a continuous variable in WII, an increment of 1 component was associated with a higher risk of dementia using the revised (HR 1.11; 95% CI 1.03, 1.19; model 2) but not the original MetS definition (HR 1.06; 95% CI 0.98, 1.14; model 2).

**Table 4:** Comparison of the original and revised definition of MetS in the association with incident dementia in the Whitehall II and UK biobank cohorts

	*N* dementia cases/Total				HR (95% CI)		*p*-value for HR comparison^c^		C-statistic (95% CI)	*p*-value for C-statistic comparison^d^
	Reference group		High-risk group		Model 1^a^	Model 2^b^				
**WHITEHALL II**										
**Median follow-up 22**.**6 (IQR 19.1**,** 27.7) years**										
Metabolic syndrome (≥ 3 cardiometabolic components)^e^										
Original MetS definition	435/4,669		87/946		1.16 (0.92, 1.47)	1.15 (0.91, 1.44)		0.19	0.573 (0.545, 0.601)	0.69
Revised MetS definition^f^	422/4,999		100/1,038		**1.26 (1.01**,** 1.57)**	1.22 (0.98, 1.52)			0.574 (0.546, 0.602)	
Increment of 1 cardiometabolic component^g^										
Original MetS definition	522/6,137				1.07 (0.99, 1.15)	1.06 (0.98, 1.14)		< 0.01	0.574 (0.547, 0.602)	0.64
Revised MetS definition^f^	522/6,137				**1.12 (1.04**,** 1.20)**	**1.11 (1.03**,** 1.19)**			0.576 (0.548, 0.604)	
**UK BIOBANK**										
**Median follow-up 13.8 (IQR 13.0**,** 14.4) years**										
Metabolic syndrome (≥ 3 cardiometabolic components)^e^										
Original MetS definition	170/103,165	248/68,721			**1.38 (1.13**,** 1.68)**	**1.31 (1.08**,** 1.60)**		**0.01**	0.630 (0.601, 0.659)	**0.04**
Revised MetS definition^h^	165/103,647	253/68,239			**1.56 (1.28**,** 1.90)**	**1.49 (1.22**,** 1.83)**			0.637 (0.608, 0.666)	
Increment of 1 cardiometabolic component^g^										
Original MetS definition	418/171,886				**1.18 (1.10**,** 1.27)**	**1.16 (1.08**,** 1.25)**		**< 0.01**	0.637 (0.608, 0.665)	0.21
Revised MetS definition^h^	418/171,886				**1.23 (1.14**,** 1.33)**	**1.21 (1.12**,** 1.30)**			0.640 (0.611, 0.668)	

MetS: metabolic syndrome; HR: hazard ratio; CI: confidence interval; IQR: interquartile range; HDL-C: high density lipoprotein-cholesterol;

^a^ Model 1: analysis adjusted for age (as time-scale), sex, education, marital status (living alone in UK Biobank), and birth cohort (5-year groups; only in Whitehall II)

^b^ Model 2: model 1 and adjustment for health-related behaviors (smoking, alcohol consumption, consumption of fruits and vegetables, and physical activity)

^c^ Using original threshold as the reference

^d^ Using original threshold as the reference

^e^ The reference group for each definition was composed of participants classified as without MetS

^f^ Contal& O’Quigley method’s thresholds for triglycerides (≥ 2.13 mmol/L) and fasting glucose (≥ 5.20 mmol/L)

^g^ The reference group for each definition was composed of participants without any MetS component

^h^ Contal& O’Quigley method’s thresholds for triglycerides (≥ 2.13 mmol/L) and glycated haemoglobin (≥ 29.8 mmol/mol)

In UKB (Table 4), the revised MetS definition (≥ 3 components; HR 1.49; 95% CI 1.22, 1.83) and 1-component increment using the revised MetS definition (HR 1.21; 95% CI 1.12, 1.30) were associated with higher risk of dementia, with stronger associations with dementia than the original MetS definition (*p* for HR comparison < 0.05). The MetS scale (0 to 5 components) had a linear association with dementia, using both the original and the revised definition for the dichotomized components in WII and UKB (Additional File 1: Table S2, and Figures S3 and S4).

Additional analyses using inverse probability weighting to account for missing data (Additional File 1: Table S3), and excluding prevalent cases of CVD at baseline (Additional File 1: Table S4) in both cohorts, yielded results similar to those in the main analyses. Analyses stratified by sex in UKB (Additional File 1: Table S5) suggested that the revised MetS definition had stronger associations with dementia in men than women although the *p*-values for interaction did not suggest differences in associations in men and women (all *p* > 0.05).

## Discussion

There are three key findings from this longitudinal study on MetS components measured before age < 60 years and incident dementia using data from two population-based cohorts in the UK. One, of the five components of MetS using the original thresholds, only waist circumference and HDL-C had a statistically significant association with incident dementia when the components were considered individually. Two, repeating the analyses using optimal thresholds identified by the Contal and O’Quigley [22] method suggests that optimized thresholds for triglycerides and fasting glucose had better performance for incident dementia than the original MetS thresholds. Three, MetS was more strongly associated with incident dementia when a revised MetS definition (using optimized thresholds for triglycerides and fasting glucose) was used. Taken together, these findings highlight the need for further assessment of the thresholds used to categorize cardiometabolic risk factors to tailor them specifically in relation to late-onset dementia.

Our study contributes to understanding the complex interplay between MetS and dementia. We opted to identify the optimal thresholds in WII and then validate our results in UKB because our focus was on midlife thresholds, and WII offered a follow-up spanning over 31 years and a larger number of incident dementia cases. We used a study design that explicitly addresses the long preclinical phase of dementia [23], by considering age at measurement of risk factors and the length of the duration of follow-up in order to circumvent reverse causation bias. All previous studies used original MetS thresholds, developed for diabetes and CVD [5, 7], to examine associations with dementia [9–13]. Dichotomizing continuous variables is widespread in medical and epidemiological research [24], serving the purposes of risk classification, establishing diagnostic criteria, and treatment recommendations. The underlying assumption of a threshold model for an exposure is the risk of disease increases after a specific cut-point is reached.

We sought to examine whether MetS component thresholds could be optimized specifically for dementia using the method proposed by Contal & O’Quigley [22]. Our findings show that none of the MetS components had a clear threshold at which the association with dementia changed in a significant manner (p-value for the Contal & O’Quigley method > 0.05). A plausible explanation for these findings is that the underlying relationship between MetS components and dementia might not be fully captured by a cut-point, probably due to linearity of associations. We undertook further analyses to examine the shape of the association between MetS components and incident dementia by comparing the linear model with the restricted cubic spline model of the association. These results show all individual components (except waist circumference in men) and MetS scale (0 to 5 components), using both the original and the revised MetS definition, to have a linear association with dementia. Results of the tests for linearity and the Contal and O’Quigley [22] method for identifying optimal thresholds are concordant apart from waist circumference in men where there was evidence of non-linearity with a U-shaped association, making it also difficult to determine a specific threshold. Taken together, these results suggest that it is difficult to establish a specific cut-point for MetS components to identify individuals at higher risk of dementia.

Although widely used, dichotomizing risk factors has long been known to be problematic due to the loss of information in the exposure variable, type I error, and non-consideration of linear associations [24]. Nevertheless, dichotomization of risk factors is widely used to allow risk stratification for primary and secondary prevention targets and treatment. Our study suggests the importance of outcome-specific identification of thresholds, along with alternate methods to validate thresholds.

Although research on MetS and dementia has used the original Mets thresholds [10–14], there is some research on alternate thresholds of individual risk factors. A previous study on identified a threshold of 130 mmHg for systolic blood pressure at 50 years through visual inspection of restricted cubic splines [15]. In line with this study, we identified an optimal threshold of 129 mmHg for systolic blood pressure at < 60 years employing robust statistical criteria and thus providing a more reliable estimate of the association with risk of dementia. However, the identified optimal thresholds for blood pressure in our analyses showed no improvement in terms of associations with risk of dementia compared with original MetS thresholds. Metabolic risk factors are thought to be important for Alzheimer’s disease and related dementias [25], making it important to identify specific risk factors and their thresholds for risk of dementia.

The association between lipids and dementia remains the subject of debate, with a recent study showing associations between several lipid fractions and dementia only in women, and only with midlife lipid levels [26], which may explain the inconsistencies for HDL-C found in our analyses. In our analyses, the identified optimal threshold for HLD-C was less restrictive in men than in women, resulting in the optimized thresholds performing worse than the original ones. A graphical representation of the relationship (**Additional File 1: Figure S2**) also indicates no clear association between HDL-C and dementia in men. The optimal threshold for triglycerides exhibited better performance for dementia risk than the original threshold, probably due to less misclassification in the high-risk group. Our findings highlight the importance of considering alternative thresholds for triglycerides in the association of MetS with late-onset dementia.

The present study sheds light on the complexity of defining MetS for associations with dementia, which involves two sequential dichotomizations. First, individual MetS components are dichotomized to classify individuals as low or high-risk. It is worth noting that although the thresholds identified by the Contal and O’Quigley’s [22] method were not statistically significant, optimal thresholds for triglycerides and fasting glucose exhibited better performance than the original thresholds. These findings align with prior research that found an increased risk of dementia among those with elevated triglycerides [10–12] and elevated fasting glucose [1, 9–12]. Second, the definition of MetS entails dichotomizing individuals based on the presence of three or more cardiometabolic abnormalities, aiming to capture the notion of a threshold effect. Previous studies on the association between MetS and late-onset dementia have mostly focused on older adults, with follow-up primarily < 10 years [10–13], and a meta-analysis of longitudinal studies found no association between MetS and dementia [14]. A potential explanation for this finding is that the original thresholds of components used to define MetS are not suitable for the risk of dementia. Our study shows that the revised MetS definition (using identified optimal thresholds for triglycerides and fasting glucose) specifically developed for dementia exhibits a fairly linear association between the number of adverse components and the risk of dementia (Additional File 1: Figures S3 and S4), with this association being stronger than that observed for the original definition. This is in line with research showing how the risk of dementia increases with the presence of even one MetS component [9, 11]. We also validated our results in an external cohort, the UKB, and found that the revised definition of MetS performed better than the original MetS definition. These findings highlight the importance of a re-examination of MetS and its components in the context of dementia risk.

Individual components of MetS have been shown to contribute to chronic low-grade inflammation, oxidative stress, and vascular dysfunction, all of them are implicated in the pathogenesis of neurodegenerative processes leading to dementia [4, 27]. Insulin resistance, a key component of MetS, impairs glucose metabolism in the brain and leads to reduced neuronal energy supply, synaptic dysfunction, and cognitive decline [28]. Dyslipidemia and hypertension can contribute to cerebral small vessel disease, microvascular dysfunction, and impaired cerebral blood flow, all associated with an increased risk of dementia [29, 30]. Furthermore, chronic low-grade inflammation associated with MetS can promote neuroinflammation, disrupt the blood-brain barrier, and trigger neuronal damage [31, 32]. Taken together, these mechanisms provide a biologically plausible explanation for the observed association between midlife MetS and late-onset dementia.

This study has several strengths such as objective measures of cardiometabolic components in midlife, long follow-up for incident dementia spanning three decades, and linkage to electronic health records for dementia ascertainment for all participants in the study. Other strengths are the use of inverse probability weighting to account for missing data at baseline due to attrition and mortality before the start of the follow-up, and the use of the UKB as a validation cohort. Our study also has some limitations. First, our analyses were only performed in white individuals, so our results cannot be generalized to other populations that may have specific, alternative optimal midlife thresholds for cardiometabolic risk factors. Second, WII and UKB study participants are likely to be healthier than the general population, although it has been previously shown risk factor-outcome associations in the WII study to be similar to those in the general population [33]. Third, as in any observational study, residual confounding may persist despite careful adjustment for covariates.

## Conclusions

The lack of curative solutions or effective treatments for dementia highlight the crucial need for identifying modifiable risk factors for developing prevention and intervention strategies. All MetS components are potentially modifiable through modification in lifestyles or pharmacological treatment, thus being important targets in reducing the risk of adverse health outcomes such as dementia. The initial purpose of MetS was to identify individuals at higher risk of type 2 diabetes and CVD, but our study shows their importance for dementia, either the revised MetS definition or as low as clinically safe, suggested by linear associations. Our findings suggest that thresholds for triglycerides and fasting glucose should be re-evaluated for prevention of late-onset dementia. Future studies should confirm whether the revised MetS definition proposed in our study is suitable for other populations.

## Electronic supplementary material

Below is the link to the electronic supplementary material.

Supplementary Material 1: Additional File 1: Table S1. Comparison of characteristics of participants included and excluded from the analyses in the Whitehall II and UK Biobank cohort studies. Table S2. Model fit statistics of the linear and restricted cubic spline models for the association of MetS and its components with incident dementia. Table S3. Comparison of the original and revised definition of MetS in the association with incident dementia in the Whitehall II and UK Biobank cohorts using inverse probability weighting to account for missing data. Table S4. Comparison of the original and revised definition of MetS in the association with incident dementia in the Whitehall II and UK Biobank cohorts excluding prevalent cases of CVD at baseline from the analyses. Table S5. Comparison of the original and revised definition of MetS in the association with incident dementia in the UK Biobank cohort, stratified by sex. Figure S1. Flow chart of sample selection in the Whitehall II and UK Biobank cohort studies. Figure S2. Associations between MetS components and incident dementia in the Whitehall II study. Figure S3. The MetS scale (scale 0 to 5) modelled as a continuous variable for the association of the number of metabolic syndrome components with incident dementia in the Whitehall II study. Figure S4. The MetS scale (scale 0 to 5) modelled as a continuous variable for the association of the number of metabolic syndrome components with incident dementia in the UK Biobank study.

## Data Availability

Whitehall II data cannot be shared publicly because of constraints dictated by the study’s ethics approval and IRB restrictions. The Whitehall II data are available for sharing within the scientific community. Researchers can apply for data access at https://www.ucl.ac.uk/epidemiology-health-care/research/epidemiology-and-public-health/research/whitehall-ii/data-sharing. The data that support the findings of this study are available from UK Biobank but restrictions apply to the availability of these data, which were used under license for the current study, and so are not publicly available. All researchers in academic, commercial and charitable settings can apply to use the UK Biobank resource for health-related research in the public interest (www.ukbiobank.ac.uk/registerapply/).

## Abbreviations

MetS: Metabolic syndrome
CVD: Cardiovascular disease
WII: Whitehall II
UKB: UK Biobank
NHS: National Health Service
HDL-C: High-density lipoprotein cholesterol
HbA1c: Glycated haemoglobin
HES: Hospital episode statistics
AIC: Akaike information criterion
BIC: Bayesian information criterion
