# Supplementary material for: Optimizing midlife metabolic syndrome thresholds for dementia: a prospective study of two UK population-based cohorts
Source: Alzheimers Res Ther. 2025 Apr 23;17:89. doi: 10.1186/s13195-025-01732-8 (PMC12016442; doi:10.1186/s13195-025-01732-8)
Supplement: Supplementary file 1 — Supplementary Material 1: Additional File 1: Table S1. Comparison of characteristics of participants included and excluded from the analyses in the Whitehall II and UK Biobank cohort studies. Table S2. Model fit statistics of the linear and restricted cubic spline models for the association of MetS and its components with incident dementia. Table S3. Comparison of the original and revised definition of MetS in the association with incident dementia in the Whitehall II and UK Biobank cohorts using inverse probability weighting to account for missing data. Table S4. Comparison of the original and revised definition of MetS in the association with incident dementia in the Whitehall II and UK Biobank cohorts excluding prevalent cases of CVD at baseline from the analyses. Table S5. Comparison of the original and revised definition of MetS in the association with incident dementia in the UK Biobank cohort, stratified by sex. Figure S1. Flow chart of sample selection in the Whitehall II and UK Biobank cohort studies. Figure S2. Associations between MetS components and incident dementia in the Whitehall II study. Figure S3. The MetS scale (scale 0 to 5) modelled as a continuous variable for the association of the number of metabolic syndrome components with incident dementia in the Whitehall II study. Figure S4. The MetS scale (scale 0 to 5) modelled as a continuous variable for the association of the number of metabolic syndrome components with incident dementia in the UK Biobank study. [file 13195_2025_1732_MOESM1_ESM.docx]

**Optimizing midlife metabolic syndrome thresholds for dementia: a prospective study of two UK population-based cohorts**

**Supplementary material**

**Table S1.** **Comparison of characteristics of participants included and excluded from the analyses in the Whitehall II and UK Biobank cohort studies.**

**Table S2. Model fit statistics of the linear and restricted cubic spline models for the association of MetS and its components with incident dementia.**

**Table S3. Comparison of the original and revised definition of MetS in the association with incident dementia in the Whitehall II and UK Biobank cohorts using inverse probability weighting to account for missing data.**

**Table S4. Comparison of the original and revised definition of MetS in the association with incident dementia in the Whitehall II and UK Biobank cohorts excluding prevalent cases of CVD at baseline from the analyses.**

**Table S5. Comparison of the original and revised definition of MetS in the association with incident dementia in the UK Biobank cohort, stratified by sex.**

**Figure S1. Flow chart of sample selection in the Whitehall II and UK Biobank cohort studies.**

**Figure S2. Associations between MetS components and incident dementia in the Whitehall II study.**

**Figure S3. The MetS scale (scale 0 to 5) modelled as a continuous variable for the association of the number of metabolic syndrome components with incident dementia in the Whitehall II study.**

**Figure S4. The MetS scale (scale 0 to 5) modelled as a continuous variable for the association of the number of metabolic syndrome components with incident dementia in the UK Biobank study.**

**Table S1. Comparison of characteristics of participants included and excluded in the analyses in the Whitehall II and UK Biobank cohort studies.**

|  | WHITEHALL II | | |  | UK BIOBANK | | |
| --- | --- | --- | --- | --- | --- | --- | --- |
|  | **Included**  **participants** | **Excluded participants** | ***p*-value** |  | **Included participants** | **Excluded participants** | ***p-*value** |
|  |  |  |  |  |  |  |  |
| N | 6,137 | 2,340 |  |  | 171,886 | 112,883 |  |
| Age, M(SD) | 55.1 (2.9) | 52.7 (4.8) | <0.01 |  | 50.7 (5.6) | 50.7 (5.6) | 0.14 |
| Sex, women | 1,756 (28.6) | 824 (35.2) | <0.01 |  | 92,444 (53.8) | 66,087 (58.5) | <0.01 |
| Education, low | 2,690 (43.8) | 1,140 (48.7) | <0.01 |  | 12,978 (7.6) | 14,282 (13.3) | <0.01 |
| Incident dementia | 522 (8.5) | 214 (9.2) | 0.35 |  | 418 (0.2) | 350 (0.3) | <0.01 |

M: mean; SD: standard deviation. Data are n (%), unless otherwise specified.

**Table S2. Model fit statistics of the linear and restricted cubic spline models for the association of MetS and its components with incident dementia.**

|  | | **Linear model^a^** | |  | **Restricted cubic spline model^a^** | |  | ***p* for**  **non-linearity^c^** |
| --- | --- | --- | --- | --- | --- | --- | --- | --- |
|  | | **AIC^b^** | **BIC^b^** |  | **AIC^b^** | **BIC^b^** |  |  |
| **WHITEHALL II** | |  |  |  |  |  |  |  |
| **Waist circumference** | |  |  |  |  |  |  |  |
|  | Men | 4980.3 | 5088.9 |  | 4977.0 | 5091.9 |  | 0.02 |
|  | Women | 2333.3 | 2426.4 |  | 2335.3 | 2433.8 |  | 0.95 |
| **Systolic blood pressure** | | 7962.6 | 8083.6 |  | 7964.3 | 8092.0 |  | 0.62 |
| **Diastolic blood pressure** | | 7963.8 | 8084.8 |  | 7965.6 | 8093.3 |  | 0.62 |
| **Triglycerides** | | 7963.7 | 8084.7 |  | 7964.7 | 8092.4 |  | 0.31 |
| **HDL-C** | |  |  |  |  |  |  |  |
|  | Men | 4980.6 | 5089.1 |  | 4980.2 | 5095.2 |  | 0.13 |
|  | Women | 2335.7 | 2428.7 |  | 2336.1 | 2434.5 |  | 0.20 |
| **Fasting glucose** | | 7958.6 | 8079.6 |  | 7960.2 | 8088.0 |  | 0.56 |
| **Original MetS definition** | | 7961.3 | 8082.3 |  | 7963.2 | 8090.9 |  | 0.78 |
| **Revised MetS definition** | | 7956.0 | 8077.0 |  | 7957.8 | 8085.6 |  | 0.67 |
| **UK BIOBANK** | |  |  |  |  |  |  |  |
| **Original MetS definition** | | 8751.0 | 8871.6 |  | 8751.9 | 8882.6 |  | 0.29 |
| **Revised MetS definition** | | 8743.7 | 8864.4 |  | 8744.1 | 8874.8 |  | 0.20 |

MetS: metabolic syndrome; AIC: Akaike Information Criterion; BIC: Bayesian Information Criterion; HDL-C: high density lipoprotein-cholesterol; UKB: UK Biobank

^a^ Model adjusted for age (as time-scale), sex, education, marital status (living alone in UK Biobank), birth cohort (5-year groups; only in Whitehall II), and health-related behaviors (smoking, alcohol consumption, consumption of fruits and vegetables, and physical activity).

^b^ Lower AIC or BIC indicates best model fit.

^c^ Non-linearity was tested by comparing the linear model with the restricted cubic spline model using the likelihood ratio test. *p*<0·05 was considered as a statistically significant non-linear relationship between the exposure and the outcome.

|  | **N dementia cases/Total** | | |  | **HR (95% CI)** | | ***p*-value for HR comparison^c^** | | **C-statistic**  **(95% CI)** | ***p*-value for**  **C-statistic comparison^d^** |
| --- | --- | --- | --- | --- | --- | --- | --- | --- | --- | --- |
|  | **Reference group** | | **High-risk group** |  | **Model 1^a^** | **Model 2^b^** |  |  |  |  |
| **WHITEHALL II** |  | |  |  |  | | | | | |
| **Median follow-up 22**.**6 (IQR 19.1, 27.7) years** |  | |  |  |  | | | | | |
| Metabolic syndrome (≥3 cardiometabolic components)^e^ |  | |  |  |  |  | |  |  |  |
| Original MetS definition | 435/4,669 | | 87/946 |  | 1.15 (0.91, 1.44) | 1.13 (0.89, 1.42) | | 0.23 | 0.573 (0.545, 0.601) | 0.71 |
| Revised MetS definition^f^ | 422/4,999 | | 100/1,038 |  | 1.23 (0.99, 1.53) | 1.19 (0.96, 1.48) | |  | 0.574 (0.547, 0.602) |  |
| Increment of 1 cardiometabolic component^g^ |  | |  |  |  |  | |  |  |  |
| Original MetS definition | 522/6,137 | | |  | 1.07 (1.00, 1.15) | 1.06 (0.99, 1.14) | | **<0.01** | 0.575 (0.547, 0.603) | 0.55 |
| Revised MetS definition^f^ | 522/6,137 | | |  | **1.12 (1.04, 1.20)** | **1.11 (1.03, 1.19)** | |  | 0.577 (0.549, 0.605) |  |
| **UK BIOBANK** |  | | |  |  |  | |  |  |  |
| **Median follow-up 13.8 (IQR 13.0, 14.4) years** |  | | |  |  |  | |  |  |  |
| Metabolic syndrome (≥3 cardiometabolic components)^e^ |  | | |  |  |  | |  |  |  |
| Original MetS definition | 170/103,165 | 248/68,721 | |  | **1.30 (1.05, 1.61)** | 1.22 (0.99, 1.52) | | **0.01** | 0.629 (0.600, 0.659) | 0.06 |
| Revised MetS definition^h^ | 165/103,647 | 253/68,239 | |  | **1.50 (1.21, 1.85)** | **1.42 (1.14, 1.76)** | |  | 0.635 (0.606, 0.664) |  |
| Increment of 1 cardiometabolic component^g^ |  | | |  |  |  | |  |  |  |
| Original MetS definition | 418/171,886 | | |  | **1.16 (1.08, 1.26)** | **1.14 (1.05, 1.23)** | | **0.01** | 0.635 (0.606, 0.664) | 0.13 |
| Revised MetS definition^h^ | 418/171,886 | | |  | **1.21 (1.11, 1.31)** | **1.18 (1.09, 1.28)** | |  | 0.638 (0.609, 0.667) |  |

**Table S3. Comparison of the original and revised definition of MetS in the association with incident dementia in the Whitehall II and UK Biobank cohorts using inverse probability weighting to account for missing data.**

MetS: metabolic syndrome; HR: hazard ratio; CI: confidence interval; IQR: interquartile range; HDL-C: high density lipoprotein-cholesterol;

^a^ Model 1: analysis adjusted for age (as time-scale), sex, education, marital status (living alone in UK Biobank), and birth cohort (5-year groups; only in Whitehall II).

^b^ Model 2: model 1 and adjustment for health-related behaviors (smoking, alcohol consumption, consumption of fruits and vegetables, and physical activity).

^c^ Using original threshold as the reference.

^d^ Using original threshold as the reference.

^e^ The reference group for each definition was composed of participants classified as without MetS.

^f^ Contal& O’Quigley method’s thresholds for triglycerides (≥2.13 mmol/L) and fasting glucose (≥5.20 mmol/L).

^g^ The reference group for each definition was composed of participants without any MetS component.

^h^ Contal& O’Quigley method’s thresholds for triglycerides (≥2.13 mmol/L) and glycated haemoglobin (≥29.8 mmol/mol).

|  | **N dementia cases/Total** | | |  | **HR (95% CI)** | | ***p*-value for HR comparison^c^** | | **C-statistic**  **(95% CI)** | ***p*-value for**  **C-statistic comparison^d^** |
| --- | --- | --- | --- | --- | --- | --- | --- | --- | --- | --- |
|  | **Reference group** | | **High-risk group** |  | **Model 1^a^** | **Model 2^b^** |  |  |  |  |
| **WHITEHALL II** |  | |  |  |  | | | | | |
| **Median follow-up 23.1 (IQR 19.1, 28.0) years** |  | |  |  |  | | | | | |
| Metabolic syndrome (≥3 cardiometabolic components)^e^ |  | |  |  |  |  | |  |  |  |
| Original MetS definition | 419/4,935 | | 82/915 |  | 1.22 (0.96, 1.54) | 1.21 (0.95, 1.53) | | 0.21 | 0.574 (0.545, 0.603) | 0.62 |
| Revised MetS definition^f^ | 407/4,842 | | 94/1,008 |  | **1.32 (1.05, 1.65)** | **1.29 (1.03, 1.61)** | |  | 0.576 (0.547, 0.604) |  |
| Increment of 1 cardiometabolic component^g^ |  | |  |  |  |  | |  |  |  |
| Original MetS definition | 501/5,850 | | |  | **1.09 (1.02, 1.18)** | **1.09 (1.01, 1.17)** | | **<0.01** | 0.576 (0.548, 0.605) | 0.42 |
| Revised MetS definition^f^ | 501/5,850 | | |  | **1.14 (1.06, 1.23)** | **1.14 (1.06, 1.23)** | |  | 0.580 (0.551, 0.608) |  |
| **UK BIOBANK** |  | | |  |  |  | |  |  |  |
| **Median follow-up 13.8 (IQR 13.0, 14.4) years** |  | | |  |  |  | |  |  |  |
| Metabolic syndrome (≥3 cardiometabolic components)^e^ |  | | |  |  |  | |  |  |  |
| Original MetS definition | 165/102,603 | 212/65,163 | |  | **1.33 (1.08, 1.63)** | **1.28 (1.04, 1.57)** | | **0.01** | 0.622 (0.591, 0.653) | 0.06 |
| Revised MetS definition^h^ | 160/103,124 | 217/64,642 | |  | **1.51 (1.23, 1.85)** | **1.46 (1.19, 1.80)** | |  | 0.629 (0.598, 0.660) |  |
| Increment of 1 cardiometabolic component^g^ |  | | |  |  |  | |  |  |  |
| Original MetS definition | 377/167,766 | | |  | **1.16 (1.07, 1.25)** | **1.14 (1.05, 1.23)** | | **<0.01** | 0.628 (0.597, 0.658) | 0.14 |
| Revised MetS definition^h^ | 377/167,766 | | |  | **1.21 (1.11, 1.31)** | **1.19 (1.10, 1.29)** | |  | 0.632 (0.601, 0.662) |  |

**Table S4. Comparison of the original and revised definition of MetS in the association with incident dementia in the Whitehall II and UK Biobank cohorts excluding prevalent cases of CVD at baseline from the analyses.**

MetS: metabolic syndrome; HR: hazard ratio; CI: confidence interval; IQR: interquartile range; HDL-C: high density lipoprotein-cholesterol;

^a^ Model 1: analysis adjusted for age (as time-scale), sex, education, marital status (living alone in UK Biobank), and birth cohort (5-year groups; only in Whitehall II).

^b^ Model 2: model 1 and adjustment for health-related behaviors (smoking, alcohol consumption, consumption of fruits and vegetables, and physical activity).

^c^ Using original threshold as the reference.

^d^ Using original threshold as the reference.

^e^ The reference group for each definition was composed of participants classified as without MetS.

^f^ Contal& O’Quigley method’s thresholds for triglycerides (≥2.13 mmol/L) and fasting glucose (≥5.20 mmol/L).

^g^ The reference group for each definition was composed of participants without any MetS component.

^h^ Contal& O’Quigley method’s thresholds for triglycerides (≥2.13 mmol/L) and glycated haemoglobin (≥29.8 mmol/mol).

**Table S5. Comparison of the original and revised definition of MetS in the association with incident dementia in the UK Biobank cohort, stratified by sex.**

|  | **MEN** | | | | |  | **WOMEN** | | | | |
| --- | --- | --- | --- | --- | --- | --- | --- | --- | --- | --- | --- |
|  | **N dementia cases/Total** | |  | **HR (95% CI)^a^** | ***p*-value for HR comparison^b^** |  | **N dementia cases/Total** | |  | **HR (95% CI)^a^** | ***p*-value for HR comparison^b^** |
|  | **Reference group** | **High-risk group** |  |  |  |  | **Reference group** | **High-risk group** |  |  |  |
| Metabolic syndrome (≥3 cardiometabolic components)^c^ |  | |  |  |  |  |  |  |  |  |  |
| Original MetS definition | 92/41,270 | 148/38,172 |  | 1.17 (0.90, 1.52) | **0.02** |  | 78/61,895 | 100/30,549 |  | **1.52 (1.13, 2.06)** | 0.20 |
| Revised MetS definition^f^ | 87/41,876 | 153/37,566 |  | **1.39 (1.06, 1.82)** |  |  | 78/61,771 | 100/30,673 |  | **1.63 (1.20, 2.21)** |  |
| Increment of 1 cardiometabolic component^e^ |  | |  |  |  |  |  |  |  |  |  |
| Original MetS definition | 240/79,442 | |  | **1.12 (1.02, 1.24)** | **<0.01** |  | 178/92,444 | |  | **1.20 (1.08, 1.34)** | 0.14 |
| Revised MetS definition^f^ | 240/79,442 | |  | **1.19 (1.07, 1.32)** |  |  | 178/92,444 | |  | **1.23 (1.10, 1.38)** |  |

MetS: metabolic syndrome; HR: hazard ratio; CI: confidence interval; IQR: interquartile range; HDL-C: high density lipoprotein-cholesterol;

^a^ Model 1: analysis adjusted for age (as time-scale), sex, education, marital status (living alone in UK Biobank), birth cohort (5-year groups; only in Whitehall II), and for health-related behaviors (smoking, alcohol consumption, consumption of fruits and vegetables, and physical activity).

^b^ Using original threshold as the reference.

^c^ The reference group for each definition was composed of participants classified as without MetS.

^d^ Contal& O’Quigley method’s thresholds for triglycerides (≥2.13 mmol/L) and fasting glucose (≥5.20 mmol/L).

^e^ The reference group for each definition was composed of participants without any MetS component.

^f^ Contal& O’Quigley method’s thresholds for triglycerides (≥2.13 mmol/L) and glycated haemoglobin (≥29.8 mmol/mol).

**Figure S1. Flow chart of sample selection in the Whitehall II and UK Biobank cohort studies.**

1. **UK BIOBANK**
2. **WHITEHALL II**


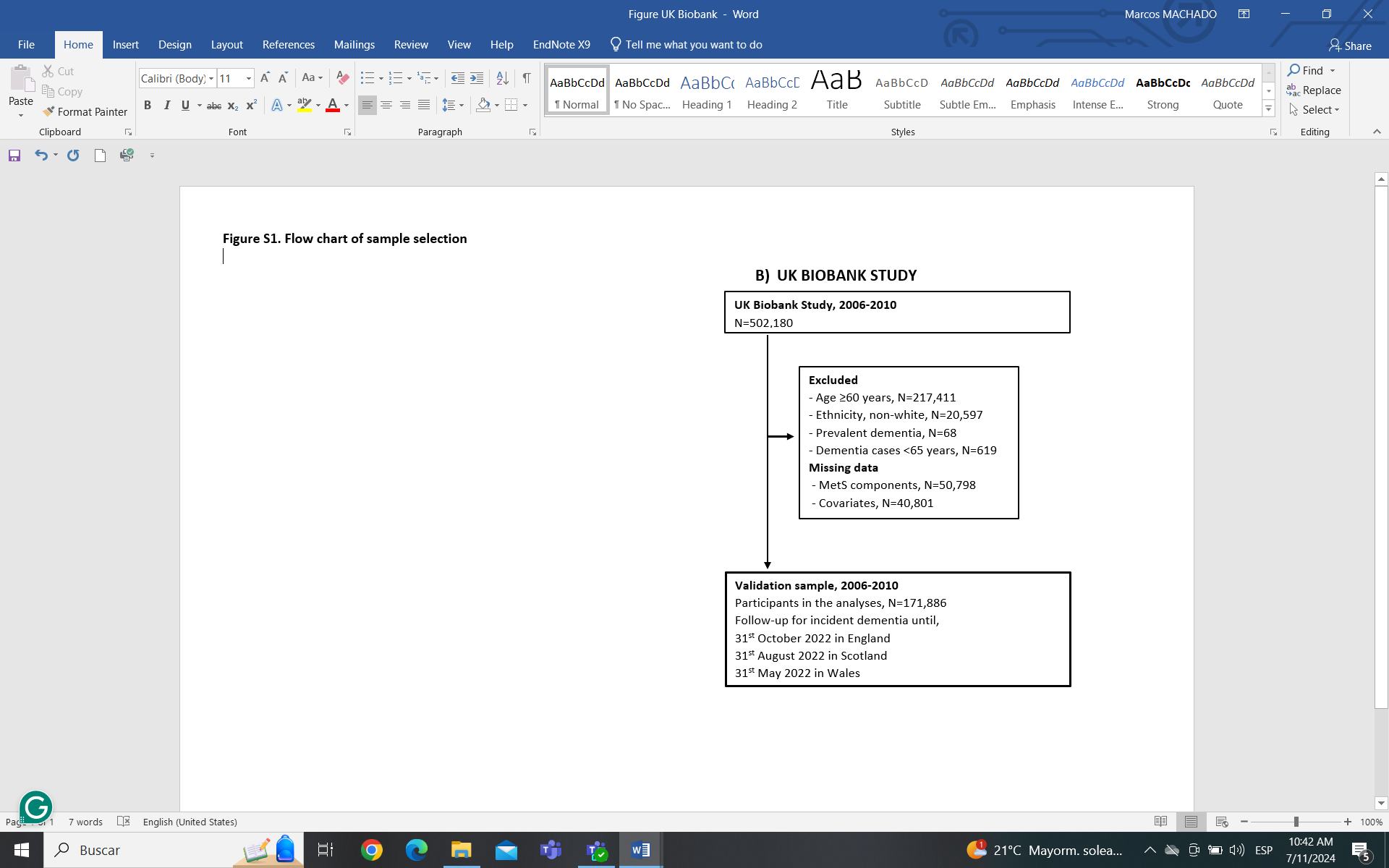

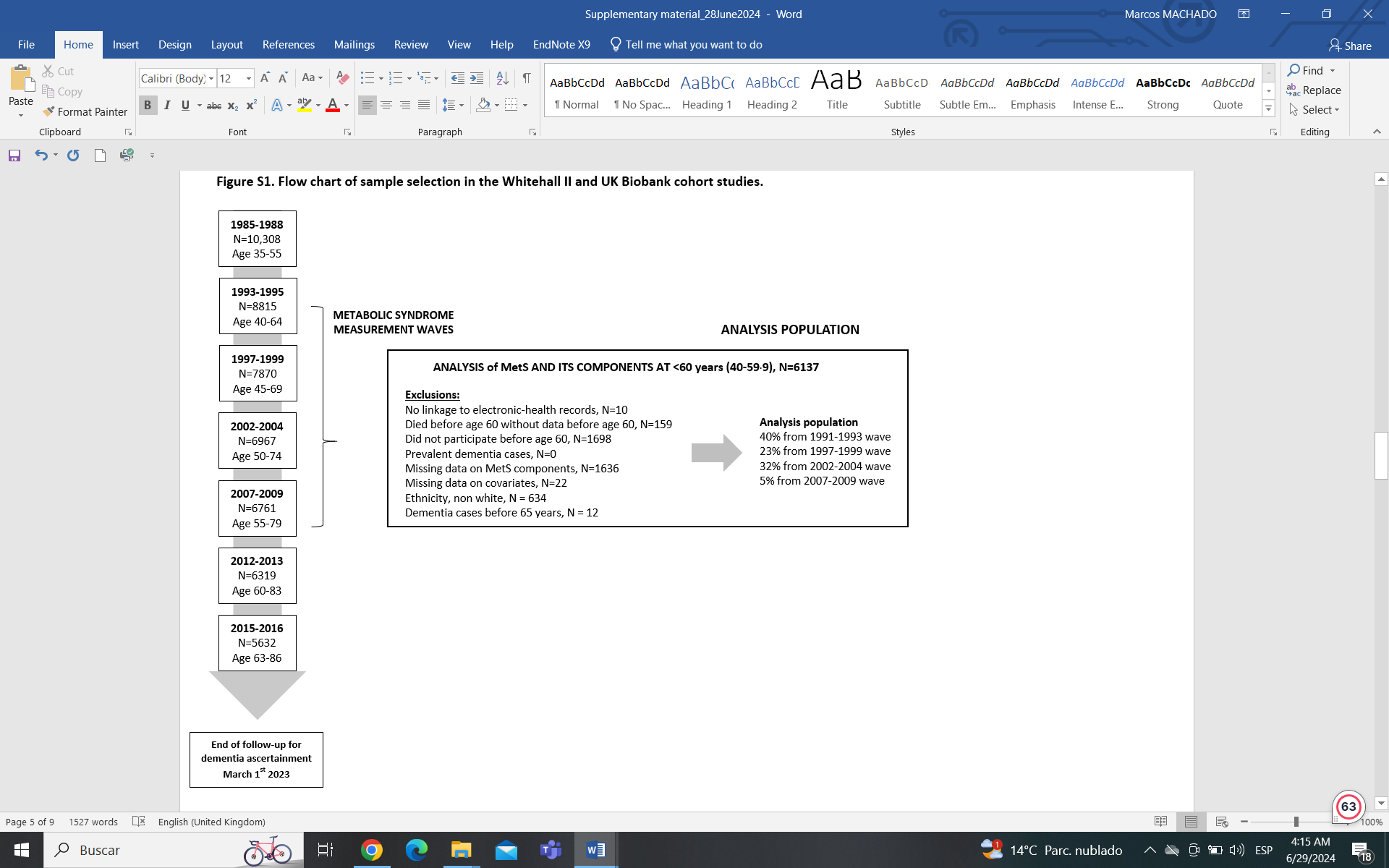


Abbreviation: MetS, metabolic syndrome

**Figure S2. Associations^a^ between MetS components and incident dementia in the Whitehall II study.^b,c^**

A) Waist circumference in men B) Waist circumference in women

C) Systolic blood pressure D) Diastolic blood pressure

E) Triglycerides F) Fasting glucose

G) HDL-C in men H) HDL-C in women

MetS: metabolic syndrome; HDL-C: high density lipoprotein cholesterol; HR: hazard ratio; CI: confidence interval.

^a^Results from Cox proportional hazard model with age as timescale, adjusted for all covariates. All figures represent the model with the best fit, using the linear model for all components except for waist circumference in men, where the restricted cubic spline model is used.

^b^Data below the 5^th^ and above the 95^th^ percentiles were excluded to avoid extreme values influencing the shape of associations. The black horizontal line is HR=1; blue line shows the estimated HR and black dash lines the 95% CI for standardized continuous MetS measures.

^c^The vertical grey line shows the original threshold for MetS, and the vertical green line that for the optimal threshold using the Contal & O’Quigley’s method.

**Figure S3. The MetS scale (scale 0 to 5) modelled as a continuous variable for the association of the number of metabolic syndrome components with incident dementia in the Whitehall II study.^a^**

**a) Metabolic syndrome components defined using original MetS definition**

**b) Metabolic syndrome components defined using revised MetS Definition^b^**

^a^Analyses using cause-specific Cox proportional hazard model with age as timescale, and adjusted for sex, education, marital status, birth cohort (5-year groups), and health-related behaviors (smoking, alcohol consumption, consumption of fruit and vegetables, and physical activity). The blue line represents the estimated HR and black dash lines represent the 95% CI. All figures represent the linear model (best fit).

^b^ Contal& O’Quigley method’s thresholds for triglycerides (≥2.13 mmol/L) and fasting glucose (≥5.2 mmol/L).

**Figure S4. The MetS scale (scale 0 to 5) modelled as a continuous variable for the association of the number of metabolic syndrome components with incident dementia in the UK Biobank study.^a^**

**a) Metabolic syndrome components defined using original MetS definition**

**b) Metabolic syndrome components defined using revised MetS Definition^b^**

**^^**

^a^Analyses using cause-specific Cox proportional hazard model with age as timescale, and adjusted for sex, education, living alone, and health-related behaviors (smoking, alcohol consumption, consumption of fruit and vegetables, and physical activity). The blue line represents the estimated HR and black dash lines represent the 95% CI. All figures represent the linear model (best fit).

^b^ Contal& O’Quigley method’s thresholds for triglycerides (≥2.13 mmol/L) and glycated haemoglobin (≥29.8 mmol/mol).
